# Perspective: Residual penile curvature correction during penile prosthesis implantation by plication in Peyronie’s patients

**DOI:** 10.1038/s41443-023-00774-6

**Published:** 2023-10-20

**Authors:** Eric Chung, Gideon Blecher

**Affiliations:** 1 AndroUrology Centre, Brisbane, QLD Australia; 2 Department of Urology, Princess Alexandra Hospital, University of Queensland, Brisbane, QLD Australia; 3 Department of Urology, Macquarie University Hospital, Sydney, NSW Australia; 4 Department of Surgery, Monash University, Melbourne, VIC Australia; 5 Department of Urology, The Alfred Hospital, Melbourne, VIC Australia

**Keywords:** Sexual dysfunction, Surgery

## Abstract

For patients with large calcified tunical plaque or severe corporal fibrosis which are likely to have a pronounced and persistent residual curvature which may not be correct by penile prosthesis implantation alone, other adjunctive manoeuvres such as penile plication and/or plaque incision with grafting may be necessary. The sequence between penile plication and penile prosthesis implantation is largely dependent on several factors such as the severity of penile curvature, the presence of (calcified) tunical plaque(s) and whether aggressive corporal dilation and subsequent penile remodelling with an inflated implant can straighten any residual penis curvature. The advantages of pre-placement of penile plication sutures prior to penile prosthesis implantation are the avoidance of inadvertent damage to the underlying penile prosthesis implant, the ability to adjust the tension on the rows of the plication sutures based on residual curvature with the device fully inflated, and potentially minimising the duration of surgery. In contrast, penile prosthesis implantation followed by penile plication to correct residual curvature, this sequence of surgery may negate the need for penile plications if penile remodelling is effective, or the residual curvature is less than 15 degrees where postoperative manual remodelling may continue to improve the penile cosmesis. When performed by expert surgeons and adhering to safe surgical principles, there is no doubt that patients will be satisfied with the outcomes and highly appreciative of the final penile cosmesis and the ensuing optimal outcomes.

## Introduction

Peyronie’s disease (PD) is typically characterised by the presence of a variety of penile deformities including penile curvature, lateral indentation, hour-glass deformity, shortening and/or hinge-defect, and in advanced cases, many males will suffer from erectile dysfunction (ED) [1–4]. Published guidelines on PD advocate various penile reconstructive strategies and it is critical to provide comprehensive counselling to set patient expectations since surgery can be associated with serious risks such as penile length loss, recurrent curvature, worsening erectile function and altered penile sensation [1–4].

For patients with combinations of penile curvature or complex deformity and coexisting ED, a penile prosthesis implant provides the definitive, reliable, and likely most effective surgical solution to simultaneously address PD and ED [5]. For patients with minor penile curvature, no further correction may be required since the insertion and subsequent cycling of the penile prosthesis implant alone may disrupt the fibrous tethering corporal scar [6–8]. It is generally accepted that a residual curvature of fewer than 20 degrees should not contribute to difficulty in sexual penetration, although some males may continue to report some forms of psychosexual distress with minor penile curvature and/or deformity [9].

While an aggressive corporal dilation and implantation of penile prosthesis alone are often sufficient to straighten minor penile curvature, those with residual curvature greater than 30 degrees may require aggressive intraoperative and postoperative penile manual modelling exercises [6–8, 10]. Presently, there is no major difference reported between the two major prosthetic devices, namely the AMS 700 CX (Boston Scientific, Marlborough, Massachusetts, USA) and Coloplast Titan (Coloplast Corp., Minneapolis, USA) prosthesis in terms of device mechanical survival and patient satisfaction rate in patients with PD receiving an inflatable penile prosthesis and remodelling [11]. However, for those with pronounced and persistent residual curvature, especially in patients with large calcified tunical plaque or severe corporal fibrosis, these will require other adjunctive manoeuvres such as penile plication and/or plaque incision with grafting, at the time of surgery [10, 12].

Penile plication is often considered a simpler adjunctive manoeuvre to incision and grafting at the time of penile prosthesis implantation since this technique is less complex, requires shorter operative time, and avoids the need for further tissue dissection including mobilisation of the neurovascular bundles, thus minimising potential complications such as sensory change in the glans penis. Presently, there are really very limited papers let alone original articles that directly compare the difference in the sequence of penile plication and penile prosthesis implantation in a true head-to-head manner. The following article evaluates the contemporary understanding of the role of penile plication in the setting of penile prosthesis implantation to address residual penile curvature and provides an important narrative perspective into the conundrum of the exact sequence of surgery by the surgeon in patients with significant penile curvature and ED.

## Surgical approach

There are various described techniques for penile plication, from traditional Nesbit corporoplasty [13] to various modified versions of Nesbit plication techniques such as Lue’s 16-dots [14], Yachia procedure (which utilises the Heineke–Mikulicz principle) [15], modification of the Duckett–Baskin tunica albuginea plication [16] or Kiel knots [17], with an emphasis on shortening the maximum convexity of the penis to correct the penile curvature. The major advantages of penile plication are its simplicity and lower complication rates compared to penile graft reconstruction [1–4]. However, this surgical technique has some disadvantages such as a loss of penile length, and penile plication does not address complex penile deformities such as lateral indentation or hour-glass deformity [1–4]. It is expected that the patient will lose approximately 1 cm of penile length for every 30 degrees of penile curvature correction [1–4, 18].

Penile plications can be incorporated into surgery when an implant is placed. A variety of skin incisions can be considered based on the surgeon’s preference and wound cosmesis [5, 19]. A subcoronal approach with circumferential degloving offers excellent access to the entire penile shaft [5]. Surgeons who perform a penoscrotal approach to penile prosthesis implantation can extend the surgical wound in the midline to dissect the penile dartos layer and access the underlying tunical albuginea at the site of penile curvature [5].

The sequence to place the penile prosthesis or plication sutures first is debatable and each of these has its own pros and cons. The decision to perform penile plication or IPP surgery is largely dependent on pre-existing penile curvature, the extent of the tunical plaque and how confident the surgeon is about the IPP surgery, whether aggressive corporal dilation and the manual prosthetic remodelling will be sufficient (Table 1). Pertinent points to discuss with prospective patients include counselling on the complexity of the surgery and potential complications, especially the postoperative loss of penile length.

**Table 1:** Advantages and disadvantages regarding the sequence of penile prosthesis implantation and plication surgery.

	Penile prosthesis implantation before penile plication surgery	Penile plication surgery before penile prosthesis implantation
Advantages	Penile remodelling may reduce the residual curvature and the need for larger plication sutures	No risk of an underlying penile prosthesis implant
Disadvantages	Risk of damage to underlying penile prosthesis cylinder Potentially longer surgical time	Potentially ‘shorter’ corporal length measurement and ‘smaller’ penile prosthesis device placement Potentially shorter penis (although the plication sutures can be adjusted)

## Penile plication sutures prior to penile prosthesis implantation

This approach allows the surgeon to perform a standard penile plication following an artificial erection test. However, instead of securing the knot of the plication sutures, a rubber shot clamp is placed on each of the sutures placed on the underlying tunica albuginea and the surgeon can adjust the tension of the sutures following the insertion and inflation of the IPP. This approach of plicating prior to implant affords the simple approach of a modified Nesbit plication technique. Furthermore, it is associated with a potentially shorter operative time since the sutures are pre-placed and can be adjusted immediately after penile prosthesis implantation to provide immediate straightness of the penis. More importantly, there is no risk of intraoperative damage to the prosthetic cylinders.

The downsides of plication before implanting include a shorter penile length and perhaps, by extension, a likely correspondingly shorter corporal length measurement for the final penile prosthesis cylinder. Hence, it is important not to tie the sutures at the time of corporal dilation and measurement. If penile remodelling with an inflated penile prosthesis implant results in a straight (or minimally residual curvature) penis, these plication sutures can be removed too.

Published literature showed this surgical approach to be effective. An early report by Mulcahy and Rowland on seven patients who underwent wedge-shaped tunical excisions following inflatable implant placement [20] reported that a straight penis and the incising or excising tunica may lead to the better burying of the suture knots. Lue and his group found that placement of ‘inverted’ loose plication sutures prior to penile prosthesis implantation will bury the plication knots to straighten the penis with the device inflated [21]. In this study with five patients who had significant 90-degree penile curvatures, no patient reported residual curvature, nor complications were noted, but the amount of length loss was not reported. In another study, Hudak et al. showed that a subset of patients who received penile plication followed by penile implant had a straight penis postoperatively, from a mean of 41 (30–55) degrees to 4 (0–10) degrees, but more than two-thirds (78%) had a reduction in penile length [22]. The same group also published a retrospective series of 18 patients who underwent plication followed by inflatable penile prostheses that demonstrated an improvement in curvature from a mean of 39 degrees (30–60) to less than 5 degrees, with high patient satisfaction rates [23]. Another single case study highlighted that pre-placement of plication suture prior to penile prosthesis implantation can allow for the surgeon to adjust the tension on the rows of the plication sutures to straighten the ‘residual curved’ penis when the penile prosthesis is fully inflated [24].

## Penile prosthesis implantation followed by penile plication

This surgical approach is advocated following the failure of penile prosthesis remodelling to straighten the penis and when residual penile curvature remains unacceptable. Placement of the necessary plication sutures risks damaging the underlying implant material. The area of curvature should be marked, and the in-situ implant should be deflated fully. If the location of curvature correction is quite distal, the implant can sometimes be milked proximally to avoid needle damage to the underlying cylinder. Plication sutures can be placed being mindful that the implant does not inadvertently slide forward. Alternatively, it is safer to deflate and remove the cylinders from the corpora body, to place the plication sutures. The surgeon will need to inflate the device first before securing the plication suture knot to straighten the penis. In this sequential surgery, the greatest concern is accidental damage to the underlying prosthetic cylinders and their tubing. Great care must be taken to avoid damaging IPP when placing the penile plication sutures.

The surgeon must be mindful of the likely reduction in penile length caused by plication sutures and the length option for the implant chosen. Malleable devices can simply be cut down to size. Inflatable devices are more nuanced. Rear-tip extenders are often used for minor length correction and in this scenario, it is recommended to consider a smaller length implant with rear-tip extenders so that the option of exchanging for a smaller length rear-tip extender remains. Alternatively, some surgeons may accurately be able to (under)estimate the expected length following penile plication. The surgeon must avoid choosing an initial inflatable implant that is too large and will not fit comfortably following the plication sutures. The benefit of this approach is that sometimes, simply by placing with aggressive corporal dilations) and inflating the implant (with or without penile modelling), the penile curvature may be largely corrected, and patients can avoid pre-emptive plication with definite length reduction. However, there remains a risk of damage to the implant, and there is an element of nuanced decision-making in choosing the implant length.

Published literature for this approach is limited. A single report from Tausch et al. compared two groups of patients where one group was known to have curvature and therefore underwent plication followed by an inflatable penile implant, while the second group was not appreciated to have a significant penile curve preoperatively and so underwent penile prosthesis with a subsequent Heineke–Mikulicz or Yachia tunical plication [25]. In both groups, all patients were straightened (to within 10 degrees residual curvature), from an average preoperative curvature of 38 and 33 degrees in groups 1 and 2, respectively.

## Discussion

The sequence between penile plication and penile prosthesis implantation is largely dependent on several factors such as the surgeon’s level of expertise, the severity of penile curvature, the presence of (calcified) tunical plaque(s) and whether aggressive corporal dilation and subsequent penile remodelling with an inflated implant can straighten any residual penis curvature. The advantages of pre-placement of penile plication sutures prior to penile prosthesis implantation are the avoidance of inadvertent damage to the underlying penile prosthesis implant, the ability to adjust the tension on the rows of the plication sutures based on residual curvature with the device fully inflated, and potentially minimising the duration of surgery. In contrast, penile prosthesis implantation followed by penile plication to correct residual curvature, this sequence of surgery may negate the need for penile plications if penile remodelling is effective, or the residual curvature is less than 15 degrees where postoperative manual remodelling may continue to improve the penile cosmesis. When performed by expert surgeons and adhering to safe surgical principles, there is no doubt that patients will be satisfied with the outcomes and highly appreciative of the final penile cosmesis and ensuing improvements in their quality of life. From the surgeon’s point of view, this dual surgical approach can be technically challenging, and the surgeon should be competent to adapt to either approach as the scenario arises. Nonetheless, the question arises whether a graft reconstruction is a more suitable option and should be performed at the time of penile prosthesis implantation to avoid penile length loss as seen in combined penile prosthesis implantation and plication.

## Conclusions

The sequence to place the penile prosthesis or plication sutures first is debatable and each of these approaches has its own advantages and disadvantages. Penile plication followed by penile prosthesis implantation avoids damage to the penile cylinders and allows adjustment of suture based on residual curvature with the penile prosthesis implant fully inflated. On the other hand, penile prosthesis implantation prior to penile plication may negate the need for penile plications if penile remodelling with an inflated device is effective or in a case where the residual curvature is less than 15 degrees. The decision to perform penile plication or IPP surgery is largely dependent on pre-existing penile curvature, the extent of the tunical plaque, how confident the surgeon is about the IPP surgery, and whether aggressive corporal dilation and manual prosthetic remodelling will be sufficient.

## Data Availability

All datasets analysed and references cited in this article are available in public domains.
